# Supplementary material for: PAX4 loss of function increases diabetes risk by altering human pancreatic endocrine cell development
Source: Nat Commun. 2023 Sep 30;14:6119. doi: 10.1038/s41467-023-41860-z (PMC10542369; doi:10.1038/s41467-023-41860-z)
Supplement: Supplementary file 1 — Supplementary Information [file 41467_2023_41860_MOESM1_ESM.pdf]

PAX4 loss of function increases diabetes risk by altering human pancreatic endocrine cell development

Supplementary Information

Supplementary Fig. 1

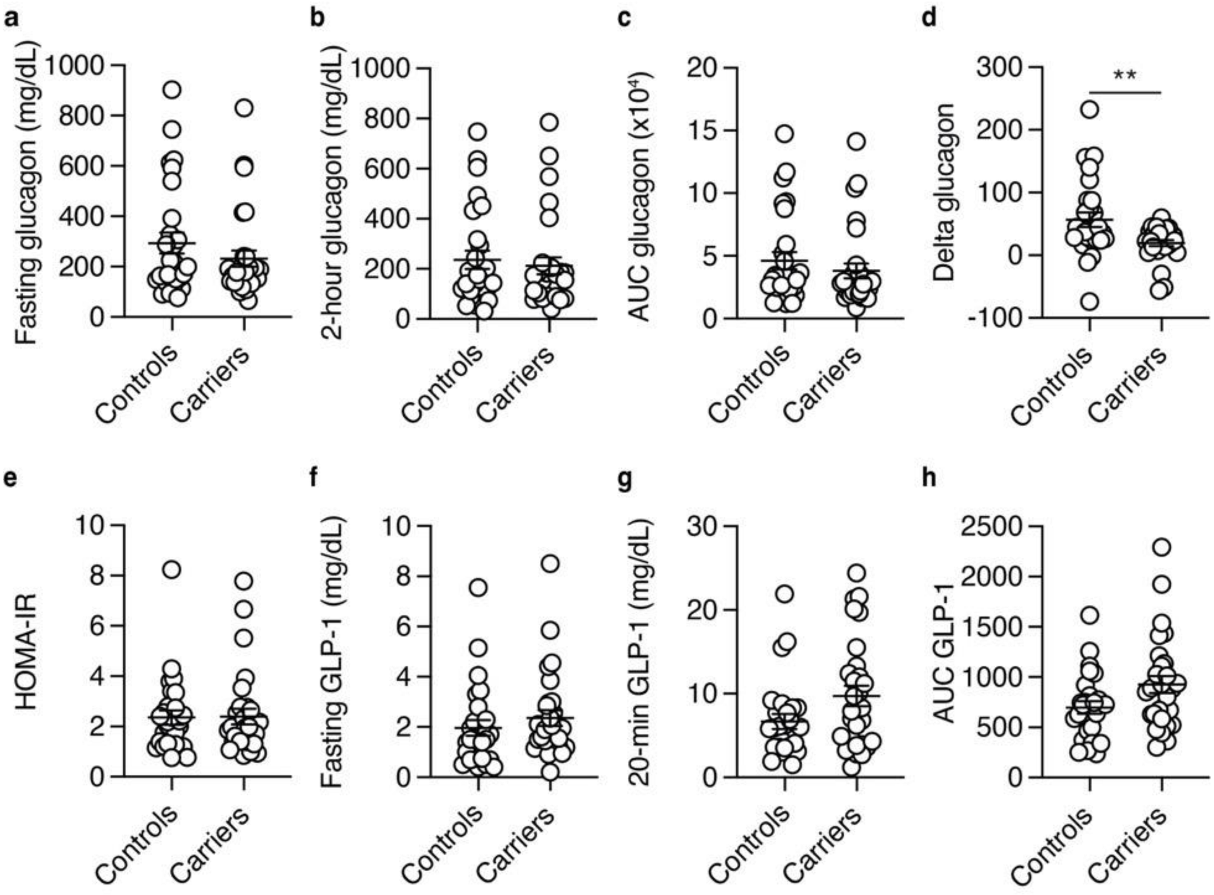

Supplementary Fig. 1 | Clinical assessment of glucagon, HOMA-IR, and GLP-1 in subjects carrying the p.Arg192His *PAX4* variant.

(**a-d**) Plasma glucagon level (mg/dL) at (**a**) fasting, (**b**) 2-hour time point, (**c**) area under the curve (AUC), and (**d**) delta glucagon during oral glucose tolerance test of subjects carrying the p.His192 allele (n=29) and p.Arg192Arg controls (n=28). (**e**) HOMA-IR measurement of p.Arg192Arg controls and p.His192 carriers during the 2-hour oral glucose tolerance test. (**f**) Fasting, (**g**) 20-min, and (**h**) AUC GLP-1 measurements during oral glucose tolerance test. Data are presented as mean±SEM. Statistical analyses were performed using two tailed unpaired Student's t-test. *p<0.05, **p<0.01. Source data is provided in the Source Data File.

Supplementary Fig. 2

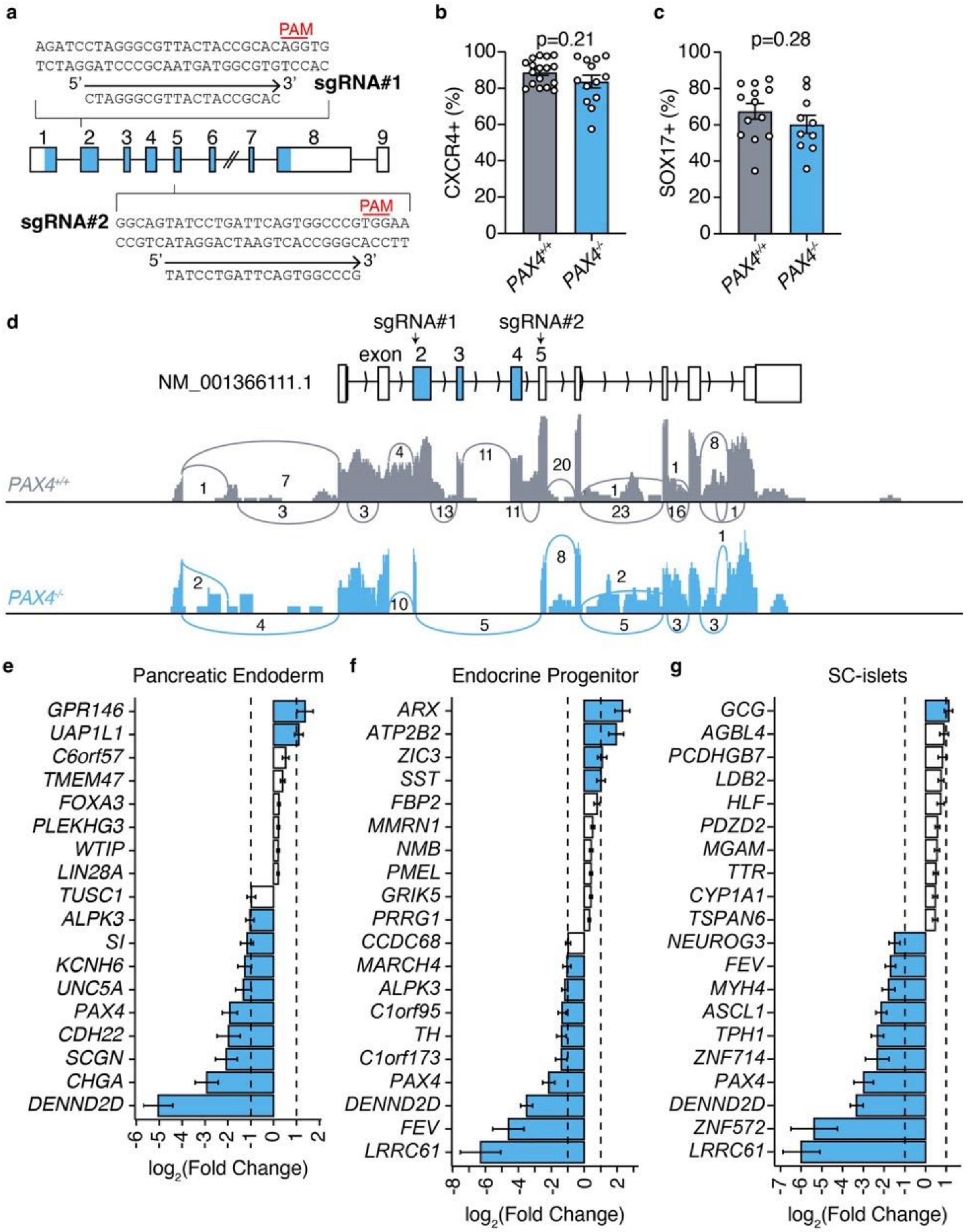

Supplementary Fig. 2 | Validation of *PAX4*$^{KO/KO}$ human induced pluripotent stem cells. (**a**) CRISPR-Cas9 genome editing strategy to generate *PAX4*$^{KO/KO}$ hiPSC line. Two sgRNAs were designed to target exon 2 (sgRNA#1) and exon 5 (sgRNA#2). PAM genomic sequence is highlighted in red. (**b-c**) Flow cytometry analyses of definitive endoderm markers (**b**) CXCR4 (n=4) and (**c**) SOX17 of wildtype (*PAX4*$^{WT/WT}$) and *PAX4*-knockout (*PAX4*$^{KO/KO}$) DE cells (n=10). (**d**) Sashimi plot of *PAX4* transcript from

Protocol A confirmed the loss of exons 2 through 5 in *PAX4*$^{KO/KO}$ lines. (**e-g**) Log_2(Fold Change) expression of top differentially expressed genes that are expressed in (**e**) pancreatic endoderm, (**f**) endocrine progenitor, and (**g**) SC-islet stages. Blue bars represent genes with a log_2(Fold Change) >1 or <-1. Each data point represents one independent experiment performed. Statistical analyses were performed by Student's t-test. Differentiation protocol A was used to derive data in Supplementary Fig. 2. Source data is provided in the Source Data File.

Supplementary Fig. 3

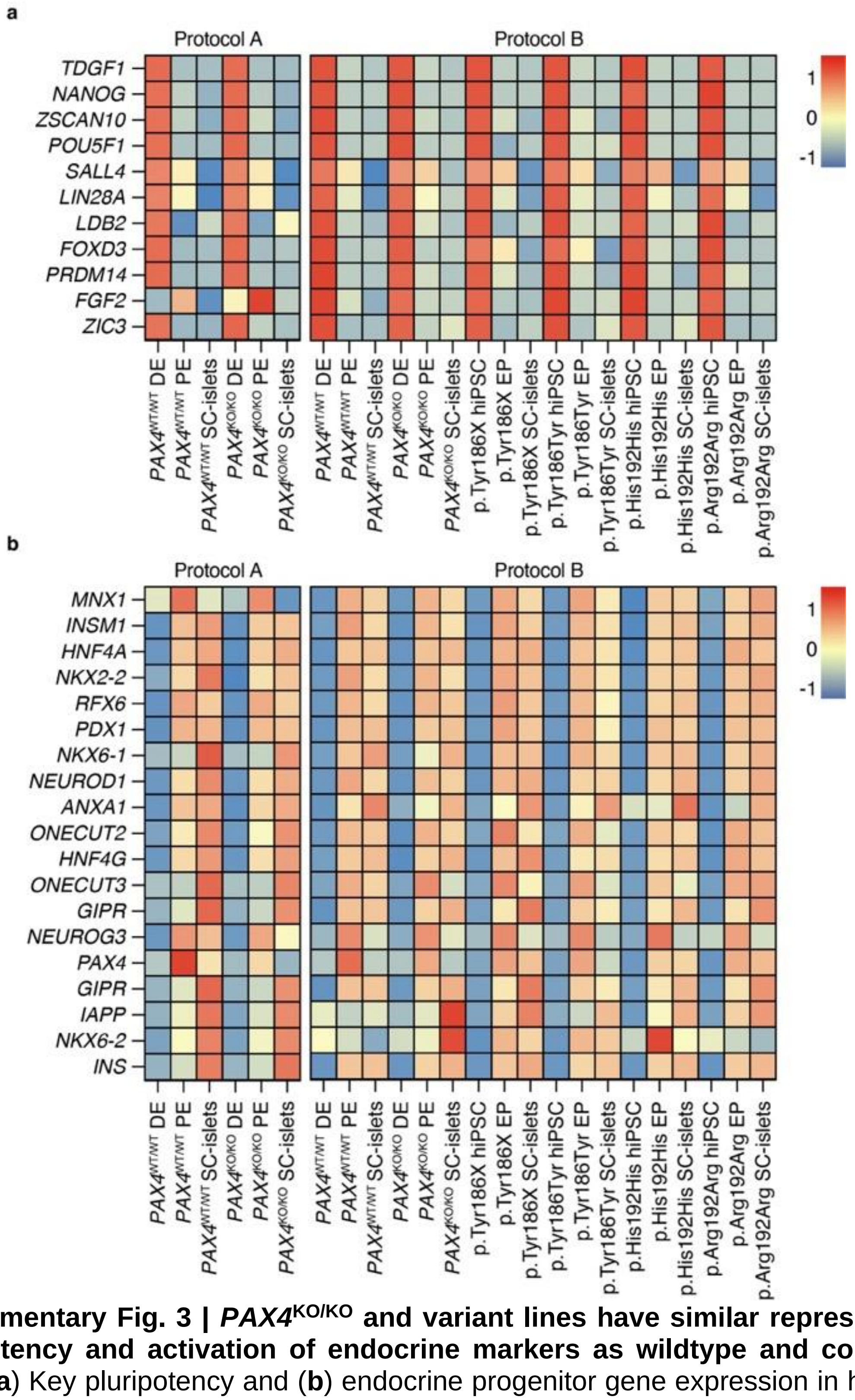

Supplementary Fig. 3 | *PAX4*$^{KO/KO}$ and variant lines have similar repression of pluripotency and activation of endocrine markers as wildtype and corrected lines. (**a**) Key pluripotency and (**b**) endocrine progenitor gene expression in hiPSCs, DE cells, EPs, and SC-islets differentiated using Protocols A and B of *PAX4* wildtype (*PAX4*$^{WT/WT}$), knockout (*PAX4*$^{KO/KO}$), *PAX4* variants (p.His192His and p.Tyr186X), and corrected (p.Arg192Arg and p.Tyr186Tyr) donor-derived hiPSC lines.

Supplementary Fig. 4

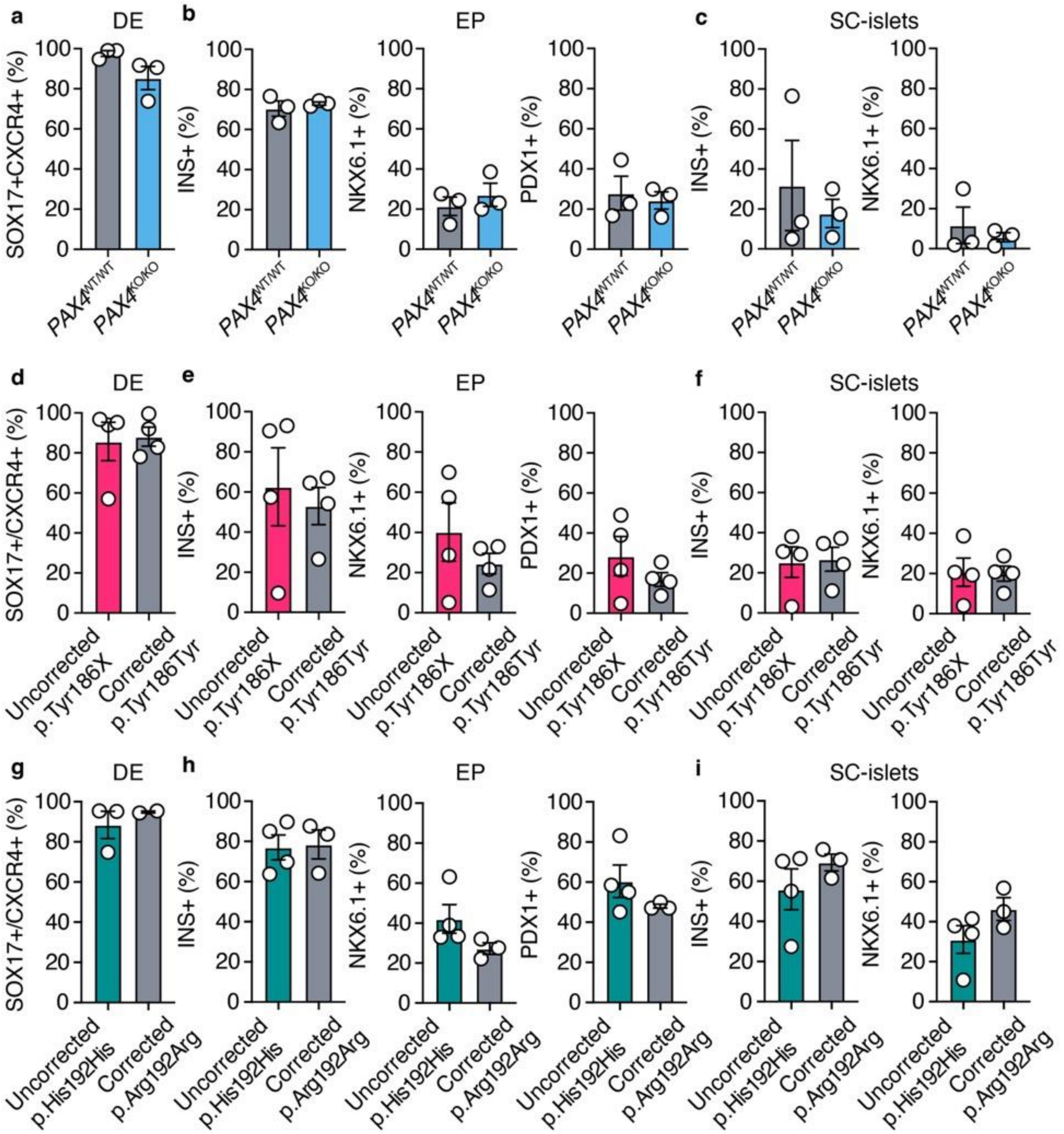

Supplementary Fig. 4 | Flow cytometry characterization of differentiation protocol B. Flow cytometry analyses of (**a**) definitive endoderm (DE) markers SOX17 and CXCR4; (**b**) endocrine progenitor (EP) markers INS, NKX6.1 and PDX1; and (**c**) islet-like cell (SC-islet) markers INS and NKX6.1 of wildtype (*PAX4*$^{WT/WT}$) and *PAX4*-knockout (*PAX4*$^{KO/KO}$) cells. Flow cytometry analyses of (**d**) DE markers SOX17 and CXCR4; (**e**) EP markers INS, NKX6.1 and PDX1; and (**f**) beta cell markers INS and NKX6.1 of p.Tyr186X and corrected p.Tyr186Tyr donor-derived hiPSC lines. Flow cytometry analyses of (**g**) DE markers SOX17 and CXCR4; (**h**) EP markers INS, NKX6.1 and PDX1; and (**i**) beta cell markers INS and NKX6.1 of p.His192His and corrected p.Arg192Arg donor-derived hiPSC lines. Each data point represents one biological independent cell line from one differentiation experiment. Data are presented as mean±SEM. Differentiation protocol B was used to derive data in Supplementary Fig. 4. Source data is provided in the Source Data File.

Supplementary Fig. 5

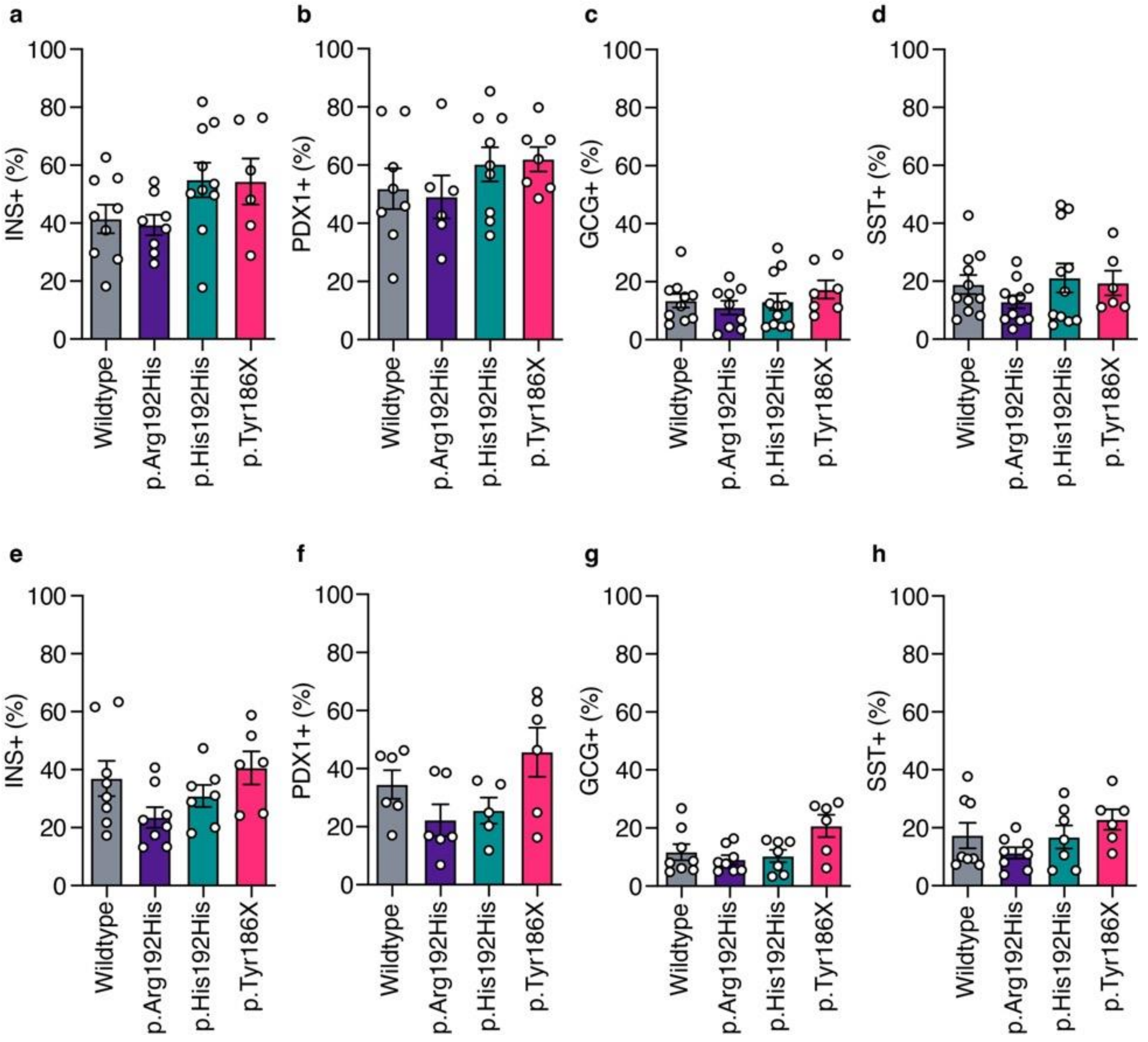

Supplementary Fig. 5 | Flow cytometry analyses on key pancreatic endocrine cell development markers to characterize endocrine progenitors and beta-like cells differentiated from donor-derived hiPSC lines. hiPSC lines derived from two wildtype, two p.Arg192His, two p.His192His and one p.Tyr186X donors were subjected to differentiation. Percentage of cells stained positive for INS, PDX1, GCG and SST on (**a-d**) D20 endocrine progenitor (EP) stage and (**e-h**) D35 islet-like cell (SC-islet) stage are presented as mean±SEM. n=5 differentiation experiments were performed. Statistical analyses were performed using one-way ANOVA. Differentiation protocol B was used to derive data in Supplementary Fig. 5. Source data is provided in the Source Data File.

Supplementary Fig. 6

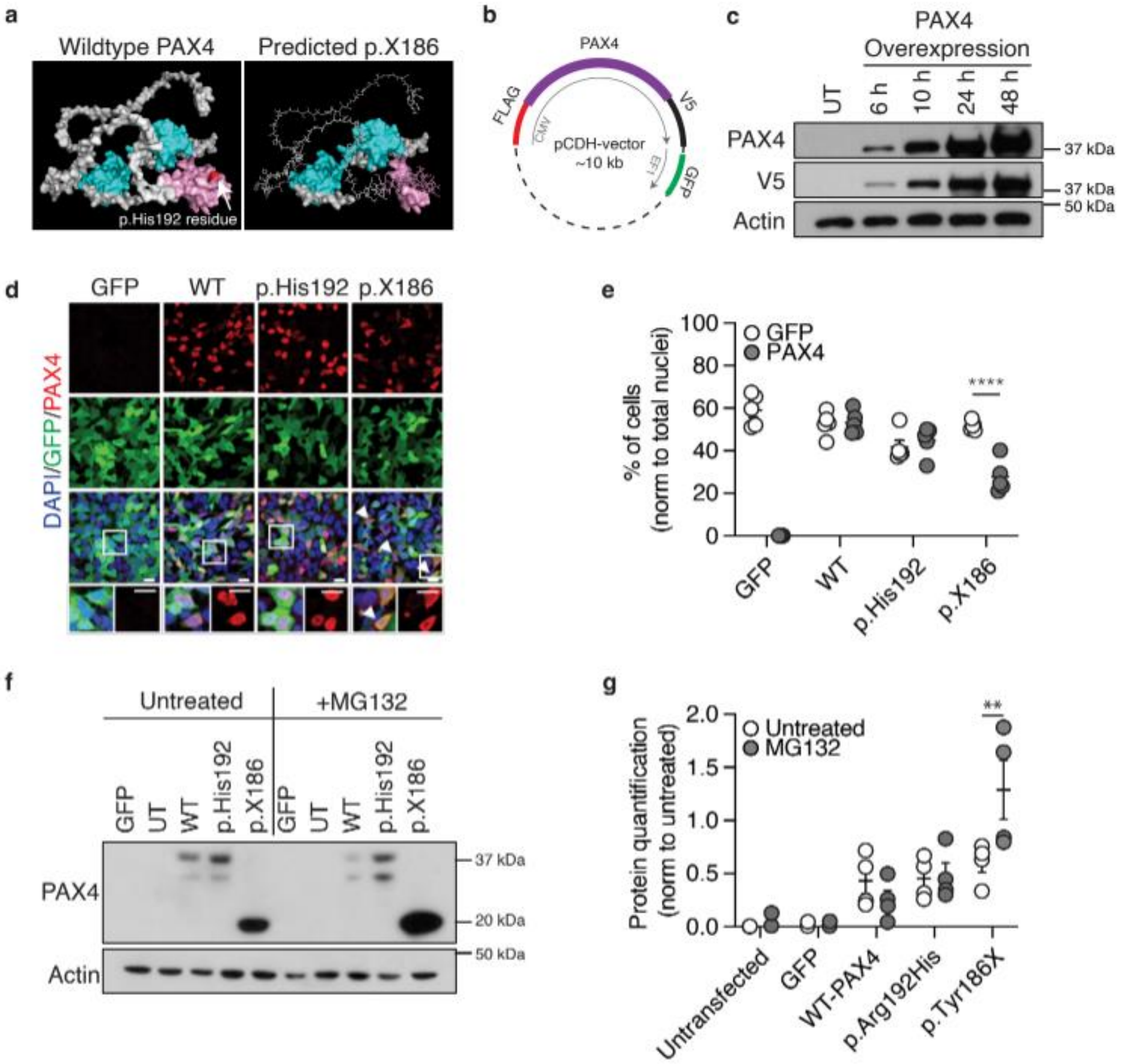

Supplementary Fig. 6 | Characterization of PAX4 and its variant proteins.
(**a**) Predicted PAX4 protein structure obtained from AlphaFold (AF-O43316-F1-model_v2). PyMOL was used for molecular visualization. Using wildtype PAX4 as template, p.X186 protein was extrapolated to demonstrate protein truncation. (**b**) Construct design for PAX4 overexpression studies. (**c**) Western blot assessment of PAX4 protein, V5 tag (~37 kD) and ACTIN loading control in AD293 cells transfected with pCDH-WT-PAX4 plasmid for 6, 10, 24 and 48 hours compared to untransfected (UT) control (n=1). (**d**) Representative immunofluorescent images of PAX4 (red, anti-PAX4 antibody), GFP (green), and nuclei (DAPI; blue) in AD293 cells following transfection of WT PAX4, p.His192, or p.X186 expressing plasmids from one experiment. Scale bar = 10 µm. (**e**) Quantification of GFP- and PAX4-expressing cells from immunofluorescence in (**d**). Percentage of cells expressing PAX4 or GFP was normalized to the total number of nuclei (DAPI). Statistical analyses were performed using two-way ANOVA and Sidak's multiple comparisons test, ****p<0.0001. (**f**) Representative image of western blot assessment and (**g**) densitometry quantification for WT PAX4, p.His192 and p.X186 was overexpressed in AD293 cells and normalized to ACTIN loading control. Cells were treated with or without 10 µM of MG132 for 24 hours posttransfection. Molecular weights of 37 kD and 20 kD correspond to WT PAX4

and p.X186 truncated protein, respectively. Anti-PAX4 antibody was used. n = 4. Statistical analyses were performed using two-way ANOVA and Sidak's multiple comparisons test, $^{**}p<0.01$. Source data is provided in the Source Data File.

Supplementary Fig. 7

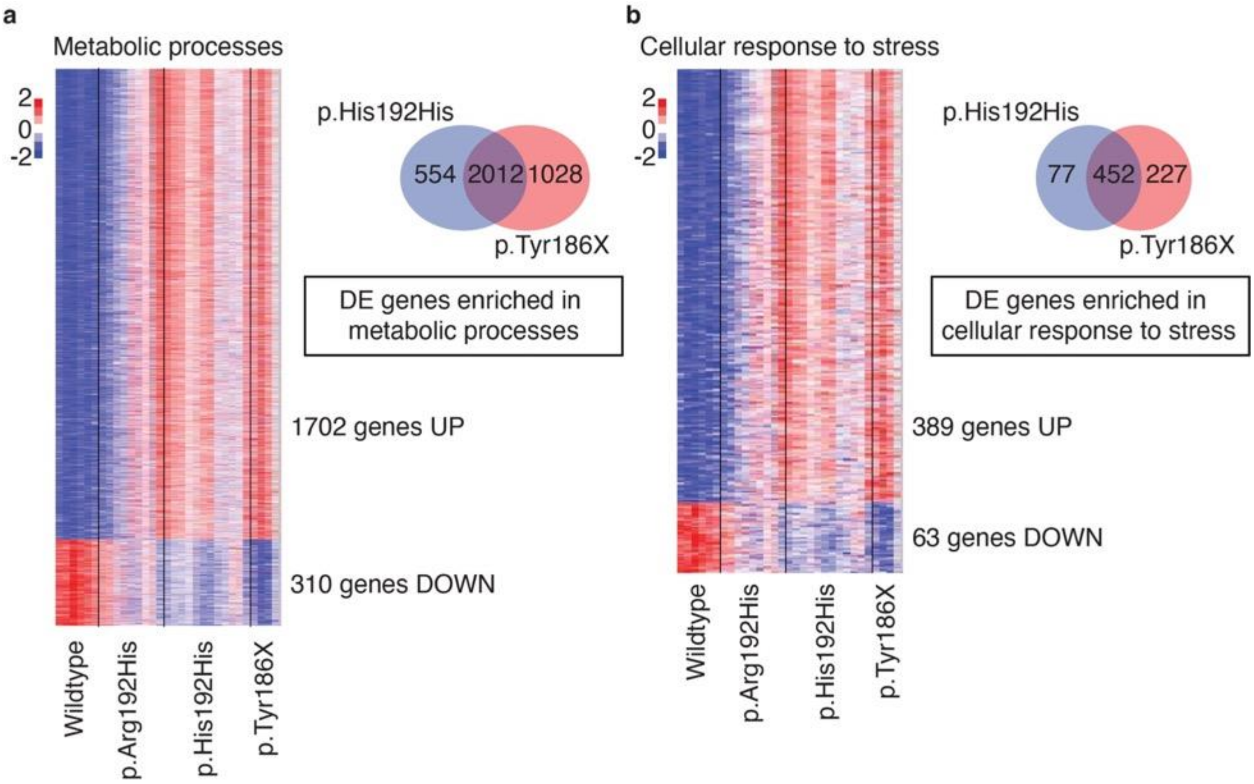

Supplementary Fig. 7 | RNA-seq revealed elevated metabolic stress in endocrine progenitors derived from donor-derived hiPSCs carrying *PAX4* variants. Targeted heatmap of differentially expressed genes in endocrine progenitors that are involved in (**a**) metabolic processes (total gene count: 2012; upregulated: 1702; downregulated: 310) and (**b**) cellular response to stress (total gene count: 452; upregulated: 389; downregulated: 63). Venn diagram illustrating differentially expressed (DE) genes enriched in GO terms (**a**) metabolic processes or (**b**) biological processes when comparing p.His192His or p.Tyr186X against *PAX4*$^{WT/WT}$ with FC < 0.5 or FC > 2. Differentiation protocol B was used to derive data in Supplementary Fig. 7.

Supplementary Fig. 8

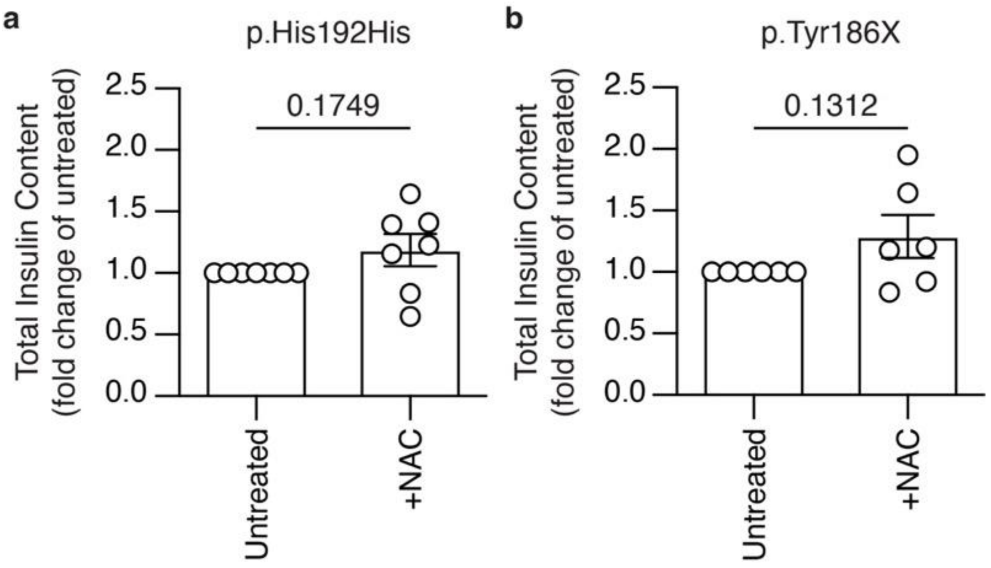

Supplementary Fig. 8 | Antioxidant treatment does not rescue the total insulin content in compromised SC-islets. Total insulin content of SC-islets treated with 10 μM antioxidant NAC from EP to SC-islet stage carrying (**a**) p.His192His or (**b**) p.Tyr186X. Each dot represents an average of technical replicates of one hiPSC line from one experiment. n=4. Data are presented as mean±SEM. Statistical analyses were performed by two-tailed Student's t-test, *p<0.05. Differentiation protocol B was used to derive data in Supplementary Fig. 8. Source data is provided in the Source Data File.

Supplementary Fig. 9

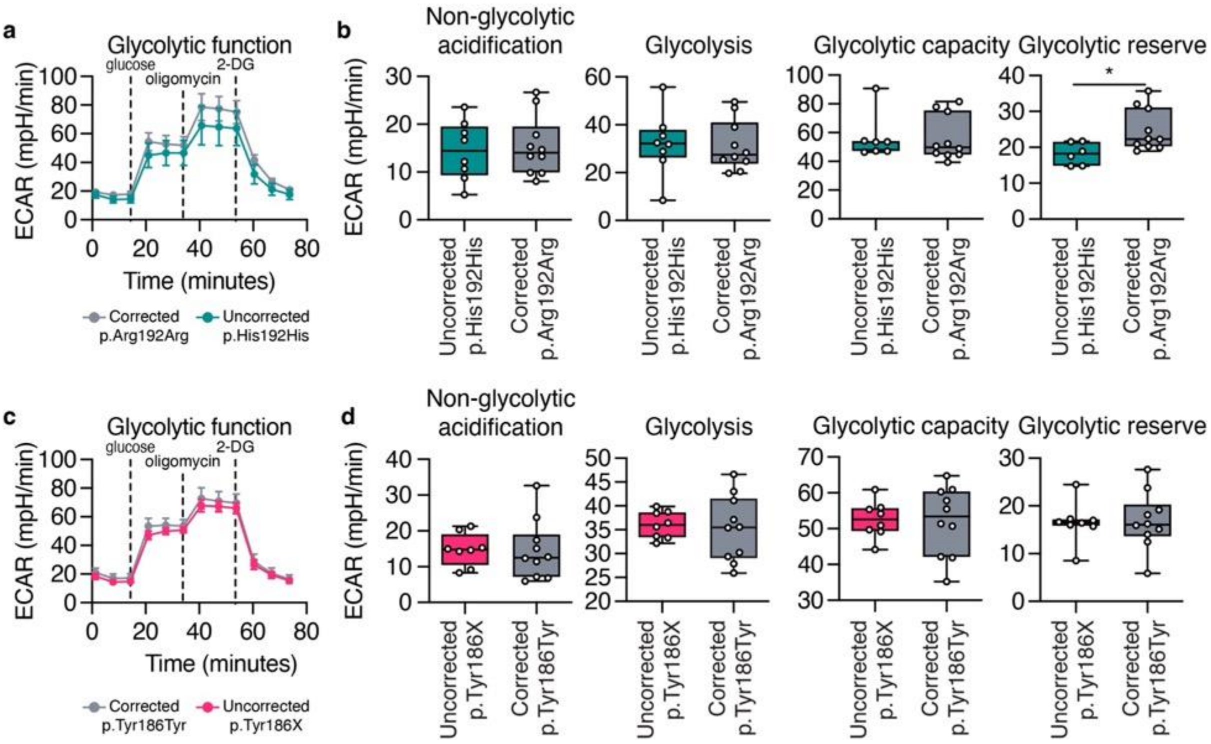

Supplementary Fig. 9 | Metabolic stress is not the main causative factor for compromised SC-islets. Glycolysis stress test on hiPSC-derived EP cells generated using protocol B. Extracellular acidification rate (ECAR) profiles of EP cells of (**a-b**) p.Arg192Arg (corrected) against p.His192His (uncorrected) and (**c-d**) p.Tyr186Tyr (corrected) against p.Tyr186X (uncorrected). Each data point represents the average measurement rate of technical replicates from one cell line. n=4 differentiation experiments were performed. Box and whisker plots illustrating median (centre), quartiles (25th and 75th percentile), maximum and minimum of all data points. Statistical analyses were performed by two-tailed Student's t-test, *$p<0.05$. Differentiation protocol B was used to derive data in Supplementary Fig. 9. Source data is provided in the Source Data File.

Supplementary Fig. 10

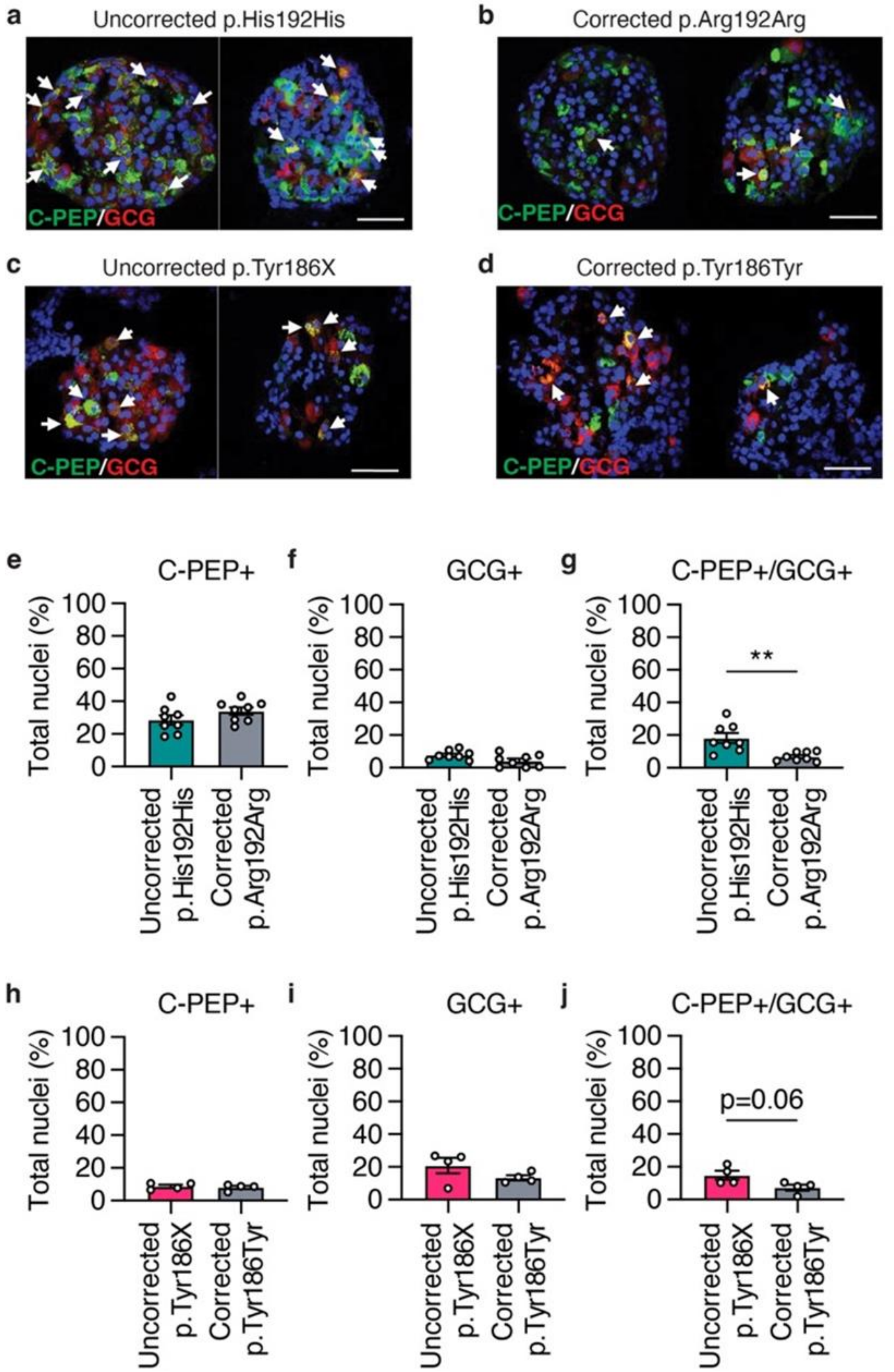

Supplementary Fig. 10 | CRISPR-correction of p.His192His or p.Tyr186X allele(s) decreased the number of polyhormonal islet-like cells. Representative immunofluorescence images of hiPSC-derived beta-like cells with C-peptide in red, glucagon in green, and nuclei in blue, (**a**) uncorrected p.His192His; (**b**) corrected

p.Arg192Arg; (**c**) uncorrected p.Tyr186X; and (**d**) corrected p.Tyr186Tyr. Arrows indicate C-PEP+/GCG+ double-positive cells. Scale bar: 50 µm. Quantification of immunofluorescence images for the percentage of cells expressing C-PEP (monohormonal), GCG (monohormonal) or C-PEP+/GCG+ (polyhormonal) in (**e-g**) uncorrected p.His192His, corrected p.Arg192Arg, (**h-j**) uncorrected p.Tyr186X; and corrected p.Tyr186Tyr islet-like cells. Differentiation protocol B was used to derive data in Supplementary Fig. 10. Data are presented as mean±SEM. All data were from one differentiation experiment. Each data point represents cell count data from one independent SC-islet. Four biological independent cell lines were used in fig. a, b, e-g. Two biological independent cell lines were used in fig. b, d, h-j. Statistical analyses were performed by two-tailed Student's t-test, $**p<0.01$. Source data is provided in the Source Data File.

Supplementary Fig. 11

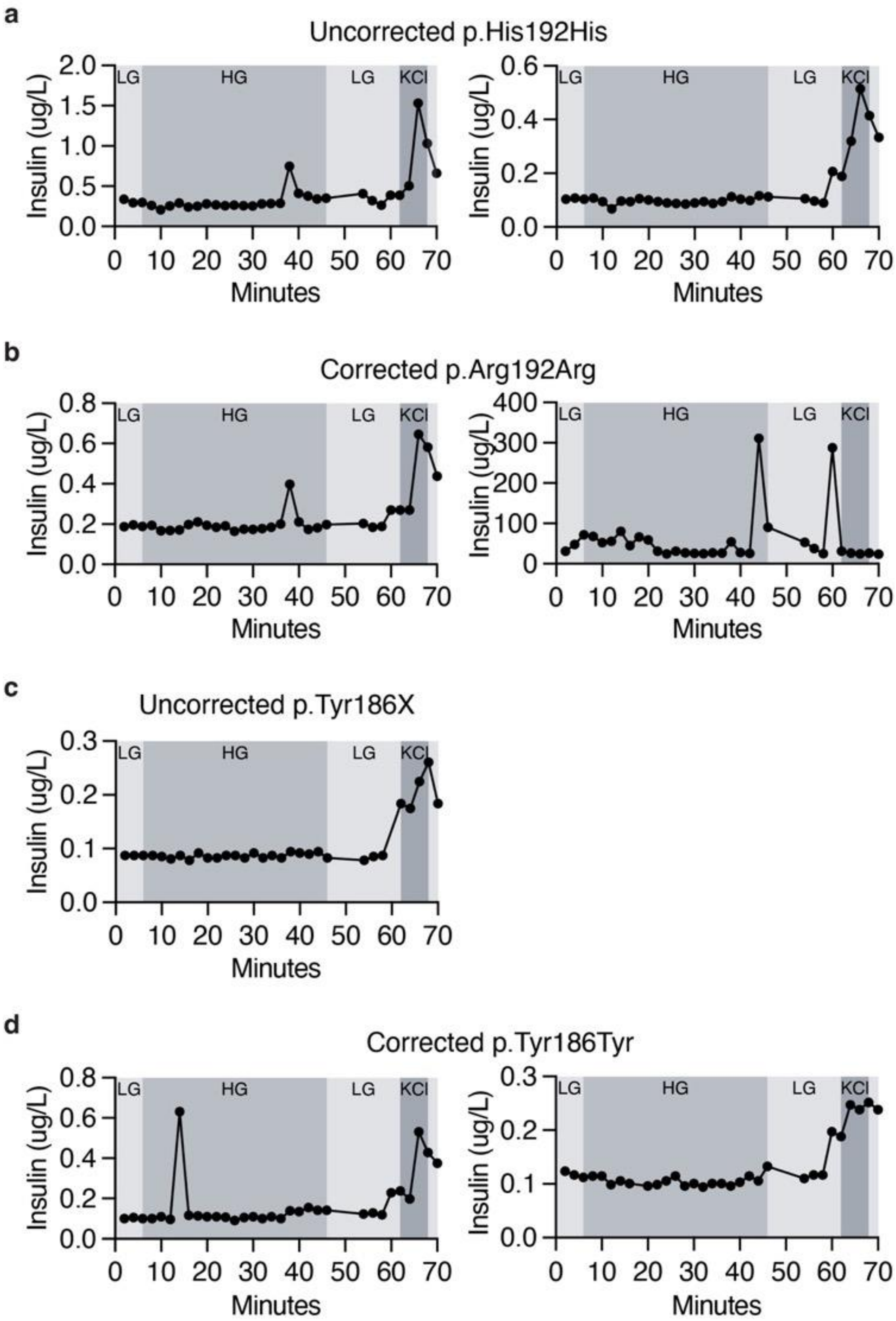

Supplementary Fig. 11 | Dynamic glucose-stimulated insulin secretion performed on beta-like cells. Donor hiPSC-derived SC-islets carrying (**a**) uncorrected p.His192His (two lines); (**b**) corrected p.Arg192Arg (two lines); (**c**) uncorrected p.Tyr186X (one line); and (**d**) corrected p.Tyr186Tyr (one line) were stimulated at 2.8 mM (6 min), 16.7 mM (40 min), 2.8 mM (16 min) and 30 mM KCL (6 min) sequentially. Each graph represents data obtained from one hiPSC line. Differentiation protocol B was used to derive data in Supplementary Fig. 11. Source data is provided in the Source Data File.

Supplementary Fig. 12. Uncropped Western blot images.

Western blot images (uncropped) for Supplementary Fig. 6c

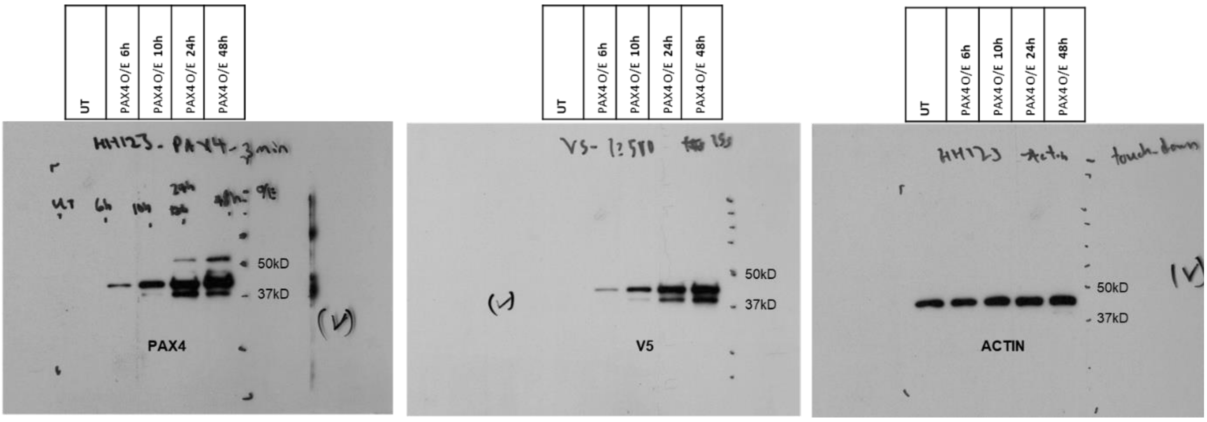

Western blot images (uncropped) for Supplementary Fig. 6f

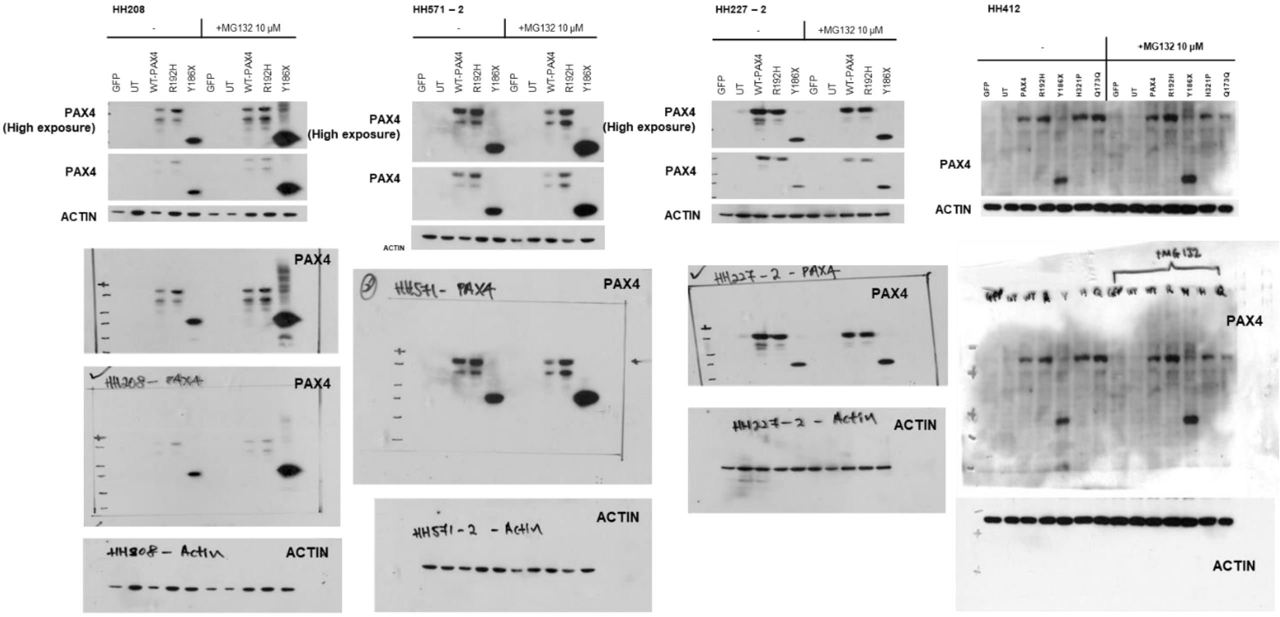

Supplementary Fig. 13. Gating strategy for all flow cytometry experiments presented in this paper.

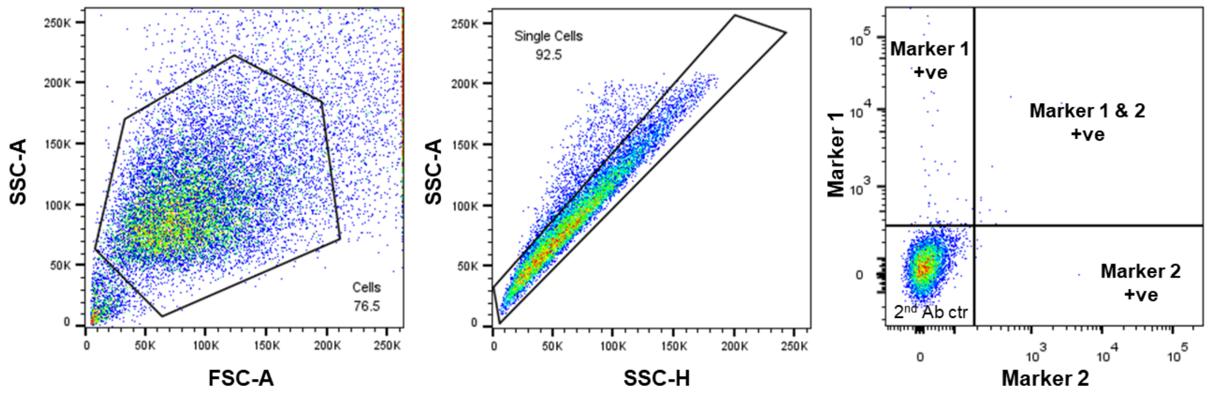

Cells were first gated on SSC-A/FSC-A, followed by gating for single cells by SSC-A/SSC-H. Cells were stained for markers of interest using primary antibodies. The percentage of positively stained cells were gated against secondary-antibody control (2º Ab ctr) staining.

Supplementary Table 1a. Gene based association analysis of *PAX4* variants with HbAc1 levels in 268,753 exomes from UKBioBank.

Variant Class	Number of Variants	P value SKAT-O	P Value Burden	P Value SKAT	CAF
pLOF	20	0.0421	0.0228	0641	2.03e-4
Missense	189	0.00683	0.0159	0.00464	0.797
Synonymous	73	0.645	0.472	0.844	0.0757

CAF, cumulative allele frequency for this gene (sum of allele frequencies). Data retrieved from https://app.genebass.org/ April 2022 (https://doi.org/10.1016/j.xgen.2022.100168).

Supplementary Table 1b. Gene based association analysis of *PAX4* variants with type 2 diabetes in 52,000 exomes from the type 2 diabetes knowledge portal.

Test	Mask	Number of Variants	Z score	P value	Odds Ratio	Standard Error	Sample Size
SKAT	LofTee	6	146476.01	0.053			43,125
Collapsing burden		6	1.70	0.088	1.21	0.111	43,125
Variable threshold		6	1.70	0.192	1.21	0.111	43,125
SKAT-optimal		6	73238.00	0.078			43,125
SKAT	16/16	6	146476.01	0.053			43,125
Collapsing burden		6	1.70	0.088	1.21	0.111	43,125
Variable threshold		6	1.70	0.192	1.21	0.111	43,125
SKAT-optimal		6	73238.00	0.078			43,125
SKAT	11/11	33	1093741.78				43,125
Collapsing burden		33	1.4796	0.139	1.05	0.034	43,125
Variable threshold		33	2.658	0.040	1.12	0.043	43,125
SKAT-optimal		33	546870.89				43,125
SKAT	**5/5**	39	24368973.87	**0.00001**			43,125
Collapsing burden		39	4.34	**0.000014**	1.06	0.013	43,125
Variable threshold		39	4.34	**0.00013**	1.06	0.013	43,125
SKAT-optimal		39	16026800.08	**0.00002**			43,125
SKAT	**5/5+ LofTee LC**	40	24419808.29	**0.00004**			43,125
Collapsing burden		40	4.43	**9.33e-6**	1.06	0.013	43,125
Variable threshold		40	4.43	**0.00008**	1.06	0.013	43,125
SKAT-optimal		40	16530425.89	**0.00002**			43,125
SKAT	**5/5 +1/5 1%**	77	226078120.98	**1.05e-31**			43,125
Collapsing burden		77	8.85	**8.47e-19**	1.06	0.007	43,125
Variable threshold		77	8.85	**1.69e1-7**	1.06	0.007	43,125
SKAT-optimal		77	126954564.88	**9.26e-31**			43,125
SKAT	**5/5 + 0/5 1%**	92	227251674.19	**5.03e-31**			43,125
Collapsing burden		92	8.91	**5.30e-19**	1.06	0.007	43,125
Variable threshold		92	8.91	**1.06e-17**	1.06	0.007	43,125
SKAT-optimal		92	129683675.78	**2.90e-31**			43,125

Data retrieved from https://t2d.hugeamp.org April 2022. Masks are described in Flannick et al Nature 2018.

Supplementary Table 2. Summary of all hiPSC lines generated and used in this study.

Origin	Genotype	Clone (SB lines)
SB_hiPSC	PAX4-wt<br>Sham ctrl	SB_Pax4 H1_B7<br>SB_Pax4 F3_H3<br>SB_Pax4 E6_E3
SB_hiPSC	PAX4-KO	SB_Pax4_KO B5_D3<br>SB_Pax4_KO D2_F3<br>SB_Pax4_KO F4_H5
**Origin**	**Genotype**	**Clone (Patient hiPSCs) - R192H**
Donor 070	p.Arg192Arg_wt	is070a<br>i070b
Donor 173	p.Arg192Arg_wt	i173b
Donor 039	p.Arg192His	is039a<br>is039b
Donor 040	p.Arg192His	is040a<br>is040b
Donor 043	p.His192His	is043a<br>i043b
Donor 181	p.His192His	is181a<br>is181b<br>is181c
Donor II-7	p.Tyr186X	iTSLa<br>iTSLb<br>iTSLc
**Origin**	**Genotype**	**Clone (Patient hiPSCs) - Y186X**
iTSLa	p.Tyr186X<br>CRISPR sham	iTsla G3<br>iTsla B3
iTSLa	p.Tyr186Tyr_wt<br>CRISPR corrected	iTsla G12<br>iTsla C12<br>iTsla D9<br>iTsla G11
**Origin**	**Genotype**	**Clone (Patient hiPSCs) - R192H**
043a	p.His192His<br>CRISPR sham	E4<br>H11
043a	p.Arg192Arg_wt<br>CRISPR corrected	B11<br>C12<br>E5<br>H9

Supplementary Table 3. Reagents used for differentiation protocols.

Reagent	Source, Catalog number
MCDB 131 media	Corning, 15-100-CV; Gibco, 10372-019
CMRL-1064 supplemented	Mediatech, 99-663-CV
Bovine serum albumin	Roche, 10775835001; Proliant, 68700
Insulin-Transferrin-Selenium-Ethanolamine	Gibco, 51500-056
L-Ascorbic acid	Sigma-Aldrich, A4544 or A8960
Heparin	Sigma-Aldrich, H3149
Human recombinant Activin A	Peprotech, 120-14; StemCell Technologies, 78001.2
CHIR 99021	Axon Medchem, 1386; Tocris, 4423
KGF/FGF-7	Peprotech, 100-19; StemCell Technologies, 78046.2
Retinoic acid	Sigma-Aldrich, R2625; WAKO, 186-01114
SANT1	Sigma-Aldrich, S4572; Santa Cruz, sc-203253
Phorbol 12,13-dibutyrate	Tocris, 4153
LDN-193189	Stemgent, 04-0074; Sigma-Aldrich, SML0559
Compound E, γ-secretase inhibitor XXI	EMD Millipore, 565789; Cayman, 15579-20
Alk5 inhibitor II	ENZO, ALX-270-445-M001
L-3,3’,5-triiodothyronine	Sigma-Aldrich, T6397; Merck Millipore, 642511
Human betacellulin	Cell Signalling, 5235SF
Alpha-Amyloid Precursor Protein Modulator	EMD Millipore, 565740
N-acetyl cysteine	Sigma-Aldrich, A9165
R428	SelleckChem, S2841
Trolox	EMD Millipore, 648471
Zinc Sulfate	Sigma-Aldrich, Z0251

Supplementary Table 4. List of cloning primers used in this study.

Cloning primers	Primer sequence (5' – 3')	Purpose
hPax4FLXbal1F	GCTCTAGAATGAACCAGCTTGGGG GGCT	To clone full-length *PAX4* sequence
hPax4FLV5Xho1R	CCGCTCGAGTTCCAAGCCATACAG TAGTGGG	
**SDM primers**	**Primer sequence (5' – 3')**	**Purpose**
Y186XSDM-F	GTGGGCAGTAGTCCTGATTCA	To create Y186X
Y186XSDM-R	TGAATCAGGACTACTGCCCAC	
R192HSDM-F	CAGTGGCCCATGGAAAGCTG	To create R192H
R192HSDM-R	CAGCTTTCCATGGGCCACTG	
**Promoter cloning primers**	**Primer sequence (5' – 3')**	**Purpose**
hINSP-1.5KpnIF	GGGGGTACCGCCTGGCTCT	*INS* gene promoter
hINSP-1EcoRVR	CCCGATATCGGCAGAAGGACA	
hGcgP-1068NheIF	CTAGCTAGCCACAGCTGGTCAATA ACAGCAA	*GCG* gene promoter
hGcgP-1 HindIIIR	CCCAAGCTTTTCTGCTGTCTTCTG GTAGTGT	
hARXPKpn-1084F	GGGGTACCAGGTGAACAGCCTCA GGGTGAAG	*ARX* gene promoter
hARXPEcoRV-7R	GGGGATATCGGCTTTTTCCCAGGG CGCAGA	
hSSTP-724FEcoRV	GGGGATATAGGGAGGGTGAGCCA GAGGT	*SST* gene promoter
hSSTP-6RHindIII	TTTAAGCTTCGCCGCGAAAGCCGA GC	
**shRNA cloning primers**	**Primer sequence (5' – 3')**	
sh*PAX*4nt315F	CGGCGGATCCTTAAGGTATCTAATCTCGAGATTAGATACC TTAAGGATCCGTTTTTG	
sh*PAX4*nt315R	ATTCAAAAACGGATCCTTAAGGTATCTAATCTCGAGATTA GATACCTTAAGGATCCG	

Supplementary Table 5. List of qPCR primers used in this study.

Targeting gene	Primer	Primer sequence (5' – 3')
ACTIN	hActinF	TTGCCGATCCGCCGCCCGTC
	hActinR	CCCATGCCCACCATCACGCCCTGG
ARX	hArxF	GGACGTCTTCACCAGGGAGGAAC
	hArxR	TCTCCCGCTTGCGCCACTT
GCG	hGcgF	ACAGCACACTACCAGAAGACAGCA
	hGcgR	TGTGCCCTGTGAATGGCGCT
INS	hInsv1-3F2	CCTGCAGGTGGGGCAGGTGGAGC
	hInsv1-3R2	CGGGTGTGGGGCTGCCTGCG
NKX6.1	hNkx6.1F	ACGCACGCCTGGCCTGTACCCC
	hNkx6.1R	CCCTCTCGGGCCCCGCCAAGTA
PAX4	hPax4F5	AGGACACGGTGAGGGTCTGGT
	hPax4R5	CAGTGGTTCCAGGGCAGGCA
PDX1	hPdx1F	CCTTCCCGGAGGGAGCCGAGCC
	hPdx1R	GTAGGCCGTGCGCGTCCGCT
SST	hSstF	GCTGCGCTGTCCATCGTCCT
	hSstR	TTGGCCAGTTCCTGCTTCCCC

Supplementary Table 6. Antibodies used in this study.

Antibody	Dilution factor	Cat#	RRID
**Primary antibody**			
Anti-beta Actin (Mouse monoclonal)	WB 1:10000	Sigma-Aldrich, A5441	AB_476744
Anti-C-PEPTIDE (Rat monoclonal)	IF 1:100	DSHB, GN-ID4	AB_2255626
Anti-CXCR4 PE-conjugated (Mouse monoclonal IgG^{2B} Clone #44717)	FC 1:10	RnD, FAB173P	AB_357083
Anti-CXCR4 APC-onjugated (Mouse Monoclonal (12G5)	FC 1:25	BD, 555976	AB_398616
Anti-FLAG M2 (Mouse monoclonal)	WB 1:1000; IF 1:100	Sigma-Aldrich, F1804	AB_262044
Anti-Glucagon Antibody (N-17) (Goat polyclonal)	IF 1:100	Santa cruz, sc-7780	AB_641025
Anti-Insulin antibody (Guinea pig polyclonal)	IF 1:100; FC 1:100	Abcam, ab7842	AB_306130
Anti-PAX4 (Goat polyclonal) *Not validated to stain endogenous protein expression	IF 1:100; WB 1:1000	RnD, AF2614	AB_2159529
Anti-SOX17 antibody (Goat polyclonal)	FC 1:100	RnD, AF1924	AB_355060
Anti-SOX2 antibody (Rabbit polyclonal)	IF 1:200	Abcam, ab97959	AB_2341193
Anti-Human SSEA-4 Antibody, Clone MC-813-70 (Mouse monoclonal)	IF 1:100	StemCell Technologies, 60062AD	AB_528477
Anti-Somatostatin Antibody (D-20) (Goat polyclonal)	IF 1:100	Santa cruz, sc-7819	AB_2302603
Anti-Human TRA-1-60 Antibody, Clone TRA-1-60R (Mouse monoclonal)	IF 1:100	StemCell Technologies, 60064AD	AB_2686905
Anti-V5 tag antibody (SV5-Pk1) (Mouse monoclonal)	IF 1:100; WB 1:1000	Abcam, ab27671	AB_471093
FC block or anti-mouse CD16/CD32 (Rat monoclonal)	FC 1:500	BD, 553141	AB_394656
Alexa Fluor® 488 anti-Human Sox17 (Mouse monoclonal Clone #P7-969)	FC 1:20	BD, 562205	AB_10893402
**Fluorophore-conjugated secondary antibody**			
Alexa Fluor® 488 anti−goat	IF 1:500; FC 1:2000	Invitrogen, A11055	AB_2534102
Alexa Fluor® 488 anti−mouse	IF 1:500; FC 1:2000	Invitrogen, A21202	AB_141607

Alexa Fluor® 488 anti−rabbit	IF 1:500; FC 1:2000	Invitrogen, A21206	AB_2535792
Alexa Fluor® 488 anti-rat	IF 1:500; FC 1:2000	Invitrogen, A21470	AB_10561519
Alexa Fluor® 594 anti−goat	IF 1:500; FC 1:2000	Invitrogen, A11058	AB_2534105
Alexa Fluor® 594 anti−Guinea pig	IF 1:500; FC 1:2000	Invitrogen, A11076	AB_141930
Alexa Fluor® 594 anti−mouse	IF 1:500; FC 1:2000	Invitrogen, A21203	AB_141633
Alexa Fluor® 594 anti−rabbit	IF 1:500; FC 1:2000	Invitrogen, A21207	AB_141637
Alexa Fluor® 647 anti-Guinea pig	IF 1:500; FC 1:2000	Jackson, 706-605-148	AB_2340476
Alexa Fluor® 647 anti-mouse	IF 1:500; FC 1:2000	Jackson, A31571	AB_162542
Alexa Fluor® 647 anti-rabbit	IF 1:500; FC 1:2000	Invitrogen, A31573	AB_2536183
Alexa Fluor® 488 Isotype	IF 1:500; FC 1:2000	BD Pharmingen, 565572	AB_2869685
**HRP-conjugated secondary antibody**			
Donkey anti-goat IgG-HRP	WB 1:10000	Santa cruz, sc-2020	AB_631728
Mouse anti-goat IgG-HRP	WB 1:5000	Santa cruz, sc-2354	AB_628490
Goat anti rabbit IgG HRP	WB 1:10000	Santa cruz, sc-2004	AB_631746
Goat anti-mouse IgG-HRP	WB 1:10000	Santa cruz, sc-2005	AB_631736
Goat anti-mouse IgG-HRP	WB 1:10000	Santa cruz, sc-2055	AB_631738
m-IgGκ BP-HRP	WB 1:5000	Santa cruz, sc-516102	AB_2687626
Mouse anti-rabbit IgG-HRP	WB 1:5000	Santa cruz, sc-2357	AB_628497

WB: Western blot; IF: Immunofluorescence; FC: Flow cytometry.
