# Supplementary material for: PAX4 loss of function increases diabetes risk by altering human pancreatic endocrine cell development
Source: Nat Commun. 2023 Sep 30;14:6119. doi: 10.1038/s41467-023-41860-z (PMC10542369; doi:10.1038/s41467-023-41860-z)
Supplement: Supplementary file 3 — Description of Additional Supplementary Files [file 41467_2023_41860_MOESM3_ESM.pdf]

Description of Additional Supplementary Files

Supplementary Data. 1

Description: RNA-seq SB hiPSCs Protocol A

Supplementary Data. 2

Description: RNA-seq SB hiPSCs Protocol B

Supplementary Data. 3

Description: RNA-seq Donor hiPSCs Protocol B

Supplementary Data. 4

Description: RNA-seq Engineered H192H Protocol B

Supplementary Data. 5

Description: RNA-seq Engineered Y186X Protocol B
