# Supplementary material for: PAX4 loss of function increases diabetes risk by altering human pancreatic endocrine cell development
Source: Nat Commun. 2023 Sep 30;14:6119. doi: 10.1038/s41467-023-41860-z (PMC10542369; doi:10.1038/s41467-023-41860-z)
Supplement: Supplementary file 9 — Reporting Summary [file 41467_2023_41860_MOESM9_ESM.pdf]

nature portfolio

Corresponding author(s): Anna Gloyn and Adrian Kee Keong Teo

Last updated by author(s): 18/9/23

Reporting Summary

Nature Portfolio wishes to improve the reproducibility of the work that we publish. This form provides structure for consistency and transparency in reporting. For further information on Nature Portfolio policies, see our Editorial Policies and the Editorial Policy Checklist.

Statistics

For all statistical analyses, confirm that the following items are present in the figure legend, table legend, main text, or Methods section.

n/a	Confirmed	
☐	☒	The exact sample size (*n*) for each experimental group/condition, given as a discrete number and unit of measurement
☐	☒	A statement on whether measurements were taken from distinct samples or whether the same sample was measured repeatedly
☐	☒	The statistical test(s) used AND whether they are one- or two-sided<br>*Only common tests should be described solely by name; describe more complex techniques in the Methods section.*
☐	☒	A description of all covariates tested
☐	☒	A description of any assumptions or corrections, such as tests of normality and adjustment for multiple comparisons
☐	☒	A full description of the statistical parameters including central tendency (e.g. means) or other basic estimates (e.g. regression coefficient) AND variation (e.g. standard deviation) or associated estimates of uncertainty (e.g. confidence intervals)
☐	☒	For null hypothesis testing, the test statistic (e.g. *F*, *t*, *r*) with confidence intervals, effect sizes, degrees of freedom and *P* value noted<br>*Give P values as exact values whenever suitable.*
☒	☐	For Bayesian analysis, information on the choice of priors and Markov chain Monte Carlo settings
☒	☐	For hierarchical and complex designs, identification of the appropriate level for tests and full reporting of outcomes
☒	☐	Estimates of effect sizes (e.g. Cohen's *d*, Pearson's *r*), indicating how they were calculated

Our web collection on statistics for biologists contains articles on many of the points above.

Software and code

Policy information about availability of computer code

Data collection: No software was used.

Data analysis:
- SPSS v28.0
- GraphPad Prism version 9
- FlowJo™ 10.6.0
- Bio-Rad CFX Maestro version 2.3
- Olympus Fluoview v3.1
- STAR v.2.5
- RStuodio version 1.4.1717
- SnapGene v6.0
- DESeq2 version 1.26.0

For manuscripts utilizing custom algorithms or software that are central to the research but not yet described in published literature, software must be made available to editors and reviewers. We strongly encourage code deposition in a community repository (e.g. GitHub). See the Nature Portfolio guidelines for submitting code & software for further information.

Data

Policy information about availability of data

All manuscripts must include a data availability statement. This statement should provide the following information, where applicable:

- Accession codes, unique identifiers, or web links for publicly available datasets
- A description of any restrictions on data availability
- For clinical datasets or third party data, please ensure that the statement adheres to our policy

Data availability: Protocol A PAX4KO/KO RNA-seq data: EGAS00001006036; Protocol B RNA-seq data: GSE203265.

Human research participants

Policy information about studies involving human research participants and Sex and Gender in Research.

Reporting on sex and gender	Sex and gender is not key consideration for our study that focused on carriers and non-carriers of PAX4 variants. However, information on sex is provided.
Population characteristics	We recruited by genotype 183 individuals without diabetes (60 heterozygous for the p.His192 allele, 2 homozygous for the p.His192 allele, and 121 controls) from two sources: 1) We recruited individuals by genotype who did not have diabetes mellitus from the multi-ethnic cohort maintained by the Saw Swee Hock School of Public Health (Singapore Population Health Studies; information accessible at https://blog.nus.edu.sg/sphs/). A subset of participants underwent whole exome sequencing as part of another study. From this sub-group, 52 individuals who were heterozygous and 2 individuals who were homozygous for the p.His192 allele were recruited to participate in this study. In addition, we recruited 72 individuals (matched for age to the carriers) who were homozygous for the p.Arg192. 2) A cross-sectional study of individuals without diabetes recruited to identify biomarkers of beta-cell function conducted at the same time in a metabolic phenotyping unit using the same study methodology provided an addition 57 individuals (8 heterozygous for the p.His192 allele and 49 individuals homozygous for the p.Arg192 allele) . All subjects were recruited and studied between 7 February 2013 and 21 August 2015. For both studies, the inclusion criteria were Chinese ethnicity, age between 21 and 80 years, non-smoker or no use of nicotine or nicotine-containing products for at least 6 months. Subjects with a known history of diabetes mellitus, screening HbA1c greater than 6.5% or fasting plasma glucose greater than 7.0 mmol/L were excluded. Subjects with weight loss greater than 5% of body weight in the preceding six months, major surgery in the last three months, a history of malignancy, estimated creatinine clearance based on the MDRD formula less than 60 mL/min, current corticosteroid use, or any clinically significant endocrine, gastrointestinal, cardiovascular, hematological, hepatic, renal, respiratory disease, or pregnancy were also excluded.
Recruitment	Participants were recruited by our clinician partners from existing research programs in Singapore. Participant selection is based on inclusion criteria, no known selection bias.
Ethics oversight	Our clinical data collection for this study was approved by the Singapore National Healthcare Group Domain Specific Review Board (NHG DSRB 2013/00937), and written informed consent was obtained from all participants. Participants were given monetary compensation for the time during which they participated in this study. The use of consented de-identified human cells (NHG DSRB 2013/00937) to generate hiPSCs for this study is covered by A*STAR IRB 2020-096. This study follows the principles of Declaration of Helsinki.<br><br>SB Ad3.1 hiPSC, Lonza CC-2511, tissue acquisition number 23447, was purchased to be used in this study. We were not responsible for obtaining approval of its use and consent.

Note that full information on the approval of the study protocol must also be provided in the manuscript.

Field-specific reporting

Please select the one below that is the best fit for your research. If you are not sure, read the appropriate sections before making your selection.

☒ Life sciences ☐ Behavioural & social sciences ☐ Ecological, evolutionary & environmental sciences

For a reference copy of the document with all sections, see nature.com/documents/nr-reporting-summary-flat.pdf

Life sciences study design

All studies must disclose on these points even when the disclosure is negative.

Sample size	Our study is not a population study, hence sample size calculation was not performed. We apply the rule of n=3 for all experiments unless otherwise stated.
Data exclusions	No data excluded for most experiment except for 1 RNAseq data from protocol B. Using UMAP (Uniform Manifold Approximation and

Data exclusions	Projection) and PCA (Principal Component Analysis) dimension reduction clustering for all four timepoints, 11 out of 164 samples were classified as outliers and excluded from clustering analyses.
Replication	All experiments were performed at least 3 times and are reproducible. Number of replicates were clearly stated in the manuscript.
Randomization	Subjects were recruited and categorized based on genotype, 62 carriers of p.Arg192His allele and 121 controls, from existing research programs in Singapore.
Blinding	This is a prospective biological study that requires no blinding. Non-personally identifiable information was the only information we used in the study.

Reporting for specific materials, systems and methods

We require information from authors about some types of materials, experimental systems and methods used in many studies. Here, indicate whether each material, system or method listed is relevant to your study. If you are not sure if a list item applies to your research, read the appropriate section before selecting a response.

Materials & experimental systems

n/a	Involved in the study
☐	☒ Antibodies
☐	☒ Eukaryotic cell lines
☒	☐ Palaeontology and archaeology
☒	☐ Animals and other organisms
☒	☐ Clinical data
☒	☐ Dual use research of concern

Methods

n/a	Involved in the study
☒	☐ ChIP-seq
☐	☒ Flow cytometry
☒	☐ MRI-based neuroimaging

Antibodies

Antibodies used

(Antibody), (Manufacturer, catalogue number), (RRID)
Anti-beta Actin (Mouse monoclonal) Sigma-Aldrich, A5441 AB_476744
Anti-C-PEPTIDE (Rat monoclonal) DSHB, GN-ID4 AB_2255626
Anti-CXCR4 PE-conjugated (Mouse monoclonal IgG2B Clone #44717) RnD, FAB173P AB_357083
Anti-CXCR4 APC-onjugated (Mouse Monoclonal (12G5) BD, 555976 AB_398616
Anti-FLAG M2 (Mouse monoclonal) Sigma-Aldrich, F1804 AB_262044
Anti-Glucagon Antibody (N-17) (Goat polyclonal) Santa cruz, sc-7780 AB_641025
Anti-Insulin antibody (Guinea pig polyclonal) Abcam, ab7842 AB_306130
Anti-PAX4 (Goat polyclonal)*Not validated to stain endogenous protein expression RnD, AF2614 AB_2159529
Anti-SOX17 antibody (Goat polyclonal) RnD, AF1924 AB_355060
Anti-SOX2 antibody (Rabbit polyclonal) Abcam, ab97959 AB_2341193
Anti-Human SSEA-4 Antibody, Clone MC-813-70 (Mouse monoclonal) StemCell Technologies, 60062AD AB_528477
Anti-Somatostatin Antibody (D-20) (Goat polyclonal) Santa cruz, sc-7819 AB_2302603
Anti-Human TRA-1-60 Antibody, Clone TRA-1-60R (Mouse monoclonal) StemCell Technologies, 60064AD AB_2686905
Anti-V5 tag antibody (SV5-Pk1) (Mouse monoclonal) Abcam, ab27671 AB_471093
FC block or anti-mouse CD16/CD32 (Rat monoclonal) BD, 553141 AB_394656
Alexa Fluor® 488 anti-Human Sox17 (Mouse monoclonal Clone #P7-969) BD, 562205 AB_10893402
Alexa Fluor® 488 anti-goat Invitrogen, A11055 AB_2534102
Alexa Fluor® 488 anti–mouse Invitrogen, A21202 AB_141607
Alexa Fluor® 488 anti–rabbit Invitrogen, A21206 AB_2535792
Alexa Fluor® 488 anti-rat Invitrogen, A21470 AB_10561519
Alexa Fluor® 594 anti–goat Invitrogen, A11058 AB_2534105
Alexa Fluor® 594 anti–Guinea pig Invitrogen, A11076 AB_141930
Alexa Fluor® 594 anti–mouse Invitrogen, A21203 AB_141633
Alexa Fluor® 594 anti–rabbit Invitrogen, A21207 AB_141637
Alexa Fluor® 647 anti-Guinea pig Jackson, 706-605-148 AB_2340476
Alexa Fluor® 647 anti-mouse Jackson, A31571 AB_162542
Alexa Fluor® 647 anti-rabbit Invitrogen, A31573 AB_2536183
Alexa Fluor® 488 Isotype BD Pharmingen, 565572 AB_2869685
Donkey anti-goat IgG-HRP Santa cruz, sc-2020 AB_631728
Mouse anti-goat IgG-HRP Santa cruz, sc-2354 AB_628490
Goat anti rabbit IgG HRP Santa cruz, sc-2004 AB_631746
Goat anti-mouse IgG-HRP Santa cruz, sc-2005 AB_631736
Goat anti-mouse IgG-HRP Santa cruz, sc-2055 AB_631738
m-IgGκ BP-HRP Santa cruz, sc-516102 AB_2687626
Mouse anti-rabbit IgG-HRP Santa cruz, sc-2357 AB_628497

Validation

All antibodies are validated by the manufacturers.
(Antibody), (application, dilution factor validated to work). WB: Western blot; IF: Immunofluorescence; FC: Flow cytometry.

Anti-beta Actin (Mouse monoclonal), WB 1:10000
Monoclonal Anti-b-Actin recognizes an epitope located on the N-terminal end of the b-isoform of actin. The antibody specifically labels b-actin in a wide variety of tissues and species using immunoblotting (42 kDa), immunofluorescent staining of cultured cell lines, and immunohistochemistry.

Anti-C-PEPTIDE (Rat monoclonal), IF 1:100
This antibody recognizes the C-peptide (aa 33-63 of proinsulin) which separates insulin B chain (aa 1-30) from insulin A chain (aa 66-86) in the proinsulin protein (minus signal peptide sequence). Stains C-peptide in mature granules and proinsulin in immature granules of islet beta-cells. The antibody does not cross-react with rodent C-peptide/proinsulin.

Anti-CXCR4 PE-conjugated (Mouse monoclonal IgG2B Clone #44717), FC 1:10
Reacts specifically with human and non-human cells expressing human CXCR4 (fusin) as detected by flow cytometry. It will also react with cells expressing feline CXCR4 but not rat CXCR4. This antibody does not cross-react with other chemokine receptors.

Anti-CXCR4 APC-onjugated (Mouse Monoclonal (12G5), FC 1:25
Reacts with fusin (CXCR4), a seven-transmembrane domain, G-protein-linked glycoprotein. Species Reactivity: Human.

Anti-FLAG M2 (Mouse monoclonal), WB 1:1000; IF 1:100
The ANTI-FLAG M2 mouse, affinity purified monoclonal antibody binds to fusion proteins containing a FLAG peptide sequence. The antibody recognizes the FLAG peptide sequence at the N-terminus, Met-N-terminus, C-terminus, and internal sites of the fusion protein.

Anti-Glucagon Antibody (N-17) (Goat polyclonal), IF 1:100
This antibody recognises epitope mapping near the N-terminus of Glucagon of human origin.

Anti-Insulin antibody (Guinea pig polyclonal), IF 1:100; FC 1:100
This antibody is raised against human insulin. It reacts with: Mouse, Rat, Human, Syrian hamster insulin.

Anti-PAX4 (Goat polyclonal) *Not validated to stain endogenous protein expression, IF 1:100; WB 1:1000
Detects human Pax4 in direct ELISAs and Western blots. In direct ELISAs, less than 5% cross-reactivity with recombinant human (rh) Pax3 is observed.

Anti-SOX17 antibody (Goat polyclonal), FC 1:100
Detects human SOX17 in direct ELISAs and Western blots. In direct ELISAs, less than 1% cross-reactivity with recombinant human (rh) SOX18 is observed.

Anti-SOX2 antibody (Rabbit polyclonal), IF 1:200
Synthetic peptide. This information is proprietary to Abcam and/or its suppliers. Reacts with: Mouse, Rat, Human SOX2.

Anti-Human SSEA-4 Antibody, Clone MC-813-70 (Mouse monoclonal), IF 1:100
This antibody reacts with rhesus, cat, chicken, dog, human, mouse, rabbit, rat. Application: cell isolation, ELISA, flow cytometry, immunocytochemistry, immunofluorescence, immunohistochemistry.

Anti-Somatostatin Antibody (D-20) (Goat polyclonal), IF 1:100
This antibody recognises epitope mapping near the C-terminus of Somatostatin of human origin.

Anti-Human TRA-1-60 Antibody, Clone TRA-1-60R (Mouse monoclonal), IF 1:100
This antibody reacts with: human, rhesus, rabbit. Application: cell isolation, flow cytometry, immunocytochemistry, immunofluorescence, immunoprecipitation, western blotting.

Anti-V5 tag antibody (SV5-Pk1) (Mouse monoclonal), IF 1:100; WB 1:1000
This antibody recognizes a small epitope, termed Pk, present on the P/V proteins of the paramyxovirus, SV5.

FC block or anti-mouse CD16/CD32 (Rat monoclonal), FC 1:500
Alexa Fluor® 488 anti-Human Sox17 (Mouse monoclonal Clone #P7-969), FC 1:20
Alexa Fluor® 488 anti–goat, IF 1:500; FC 1:2000
Alexa Fluor® 488 anti–mouse, IF 1:500; FC 1:2000
Alexa Fluor® 488 anti–rabbit, IF 1:500; FC 1:2000
Alexa Fluor® 488 anti-rat, IF 1:500; FC 1:2000
Alexa Fluor® 594 anti–goat, IF 1:500; FC 1:2000
Alexa Fluor® 594 anti–Guinea pig, IF 1:500; FC 1:2000
Alexa Fluor® 594 anti–mouse, IF 1:500; FC 1:2000
Alexa Fluor® 594 anti–rabbit, IF 1:500; FC 1:2000
Alexa Fluor® 647 anti-Guinea pig, IF 1:500; FC 1:2000
Alexa Fluor® 647 anti-mouse, IF 1:500; FC 1:2000
Alexa Fluor® 647 anti-rabbit, IF 1:500; FC 1:2000
Alexa Fluor® 488 Isotype, IF 1:500; FC 1:2000
Donkey anti-goat IgG-HRP, WB 1:10000
Mouse anti-goat IgG-HRP, WB 1:5000
Goat anti rabbit IgG HRP, WB 1:10000
Goat anti-mouse IgG-HRP, WB 1:10000
Goat anti-mouse IgG-HRP, WB 1:10000
m-IgGκ BP-HRP, WB 1:5000
Mouse anti-rabbit IgG-HRP, WB 1:5000

Eukaryotic cell lines

Policy information about cell lines and Sex and Gender in Research

Cell line source(s)	AD-293 line Agilent 240085 RRID:CVCL_KA63<br>293FT Invitrogen, R70007, RRID:CVCL_6911<br>EndoC-"H1 line Univercell Biosolutions EndoC-"H1 RRID:CVCL_L909<br>Alpha TC clone 9 mouse pancreatic adenoma cells (αTC1.9) (ATCC, CRL-2350™) RRID:CVCL_0150<br>SB Ad3.1 hiPSC, Lonza CC-2511, tissue acquisition number 23447<br>All donor derived hiPSC lines were generated in this study
Authentication	Authentication of hiPSCs generated in this study were performed using karyotyping. All commercially available cell lines were not authenticated.
Mycoplasma contamination	All cell lines were routinely tested negative for mycoplasma.
Commonly misidentified lines<br>(See ICLAC register)	No commonly misidentified cell lines were used.

Flow Cytometry

Plots

Confirm that:

☒ The axis labels state the marker and fluorochrome used (e.g. CD4-FITC).

☒ The axis scales are clearly visible. Include numbers along axes only for bottom left plot of group (a 'group' is an analysis of identical markers).

☒ All plots are contour plots with outliers or pseudocolor plots.

☒ A numerical value for number of cells or percentage (with statistics) is provided.

Methodology

Sample preparation	DE cells generated with Protocol A were collected at the end of Stage 1 using Accutase. For extracellular staining, cells were washed twice with 1X Flow Cytometry Staining Buffer (RnD, FC001). Cells were blocked in Flow Cytometry Staining Buffer with FC block for 5 min before adding antibody for 45 min. Cells were then washed twice with Flow Cytometry Staining Buffer. For intracellular staining, cells were fixed using BD CytoFix Buffer (BD Biosciences, 554655) for 20 min on ice before washing twice with PBS. Using BD Perm/Wash Buffer (BD Biosciences, 554723), fixed cells were permeabilized for 30 min on ice, washed three times, and incubated with antibody for 1 hour at 4 oC before a final wash step in PBS.<br>DE, EPs and BLCs were collected on D3, D20 and D35, respectively, following differentiation with Protocol B before being dissociated into single cells using TrypLE™ Express (Gibco, 12605-010). Cells were passed through a 40 µm cell strainer and single cells were fixed with 4% PFA for 20 min on ice. Antigen blocking and cell permeabilization were performed using DPBS supplemented with 5% FBS (HyClone, SV30160.03) and 0.1% Triton-X-100 for 30 min on ice. Cells were stained with primary antibodies for 1 hour at room temperature. The cells were then washed three times with DPBS and incubated with corresponding secondary antibodies for 1 hour at room temperature.
Instrument	SH800 Cell Sorter (Sony), BD® LSR II Flow Cytometer (BD Biosciences)
Software	FlowJo™ 10.6.0
Cell population abundance	No cell sorting was performed in this study.
Gating strategy	Cells were first gated on SSC-A/FSC-A, followed by gating for single cells by SSC-A/SSC-H. Cells were stained for markers of interest using primary antibodies. The percentage of positively stained cells were gated against secondary-antibody control staining.

☒ Tick this box to confirm that a figure exemplifying the gating strategy is provided in the Supplementary Information.
